# The protocol for assessing olfactory working memory capacity in mice

**DOI:** 10.1002/brb3.2703

**Published:** 2022-07-18

**Authors:** Li‐Xin Jiang, Geng‐Di Huang, Hua‐Li Wang, Chen Zhang, Xin Yu

**Affiliations:** ^1^ Peking University Institute of Mental Health (Sixth Hospital) Beijing China; ^2^ National Clinical Research Center for Mental Disorders & NHC Key Laboratory of Mental Health (Peking University) Beijing China; ^3^ Beijing Municipal Key Laboratory for Translational Research on Diagnosis and Treatment of Dementia Beijing China; ^4^ Peking University Shenzhen Graduate School Shenzhen China; ^5^ Shenzhen Kangning Hospital & Shenzhen Mental Health Center Shenzhen China; ^6^ Capital Medical University, Youanmenwai Beijing China

**Keywords:** 5×FAD mice, odor, working memory capacity

## Abstract

**Background:**

Working memory capacity (WMC) is the ability to maintain information over a few seconds. Although it has been extensively studied in healthy subjects and neuropsychiatric patients, few tasks have been developed to measure such changes in rodents. Many procedures have been used to measure WM in rodents, including the radial arm maze, the WM version of the Morris swimming task, and various delayed matching and nonmatching‐to‐sample tasks. It should be noted, however, that the memory components assessed in these procedures do not include memory capacity.

**Methods:**

We developed an olfactory working memory capacity (OWMC) paradigm to assess the WMC of 3‐month‐old 5×FAD mice, a mouse model of Alzheimer's disease. The task is divided into five phases: context adaptation, digging training, rule learning for nonmatching to a single sample odor (NMSS), rule learning for nonmatching to multiple sample odors (NMMS), and capacity testing.

**Results:**

In the NMSS rule‐learning phase, there was no difference between wild‐type (WT) mice and 5×FAD mice in the performance correct rate, correct option rate, and correct rejection rate. The WT mice and 5×FAD mice showed similar memory capacity in the NMMS rule‐learning phase. After capacity test, we found that the WMC was significantly diminished in 5×FAD mice. As the memory load increased, 5×FAD mice also made significantly more errors than WT mice.

**Conclusion:**

The OWMC task, based on a nonmatch‐to‐sample rule, is a sensitive and robust behavioral assay that we validated as a reliable method for measuring WMC and exploring different components of memory in mice.

## 1 INTRODUCTION

Working memory (WM) refers to the ability to briefly maintain and manipulate goal‐related information to guide upcoming actions (D'Esposito, 2007; Hasselmo & Stern, 2006; Lundqvist et al., 2018; Miller et al., 2018). It is associated with persistent neural activity in multiple brain regions and is considered a core cognitive process that supports a series of behaviors, from perception to trouble shooting and action control (Constantinidis et al., 2018; Ma et al., 2014; Zylberberg & Strowbridge, 2017). WM is a basic function by which we can get rid of reflexive input‐output reactions and thus control our own thoughts (Badre & Badre, 2015; Miller, 2001; Vogel & Machizawa, 2004). WMC, a critical component of WM, is the ability to retain information for a few seconds (Constantinidis & Klingberg, 2016). The WMC is limited, and a restricted amount of information or number of items is actively retained in WM. One of the major restrictions of human cognition is the limited amount of information that can be held in WM (Cowan, 2001; Ga, 1956). Individual differences in WMC have also been associated with changes in certain important abilities, including attention control, nonverbal reasoning ability, and academic performance (Conway et al., 2003; Kane et al., 2007; Klingberg, 2010). WM deficits are apparent in older individuals, who are susceptible to cognitive deterioration (Grady, 2012; Park & Reuter‐Lorenz, 2009; Rypma & D'Esposito, 2000). A lower WMC is also a feature of many clinical populations, including individuals with Alzheimer's disease (AD) (Belleville et al., 2007; Huntley & Howard, 2010; Stopford et al., 2012), schizophrenia (Fleming et al., 2002; Fleming et al., 1997; Gold et al., 2010), attention deficit‐hyperactivity disorder (Martinussen et al., 2005; Willcutt et al., 2005). Measures of WMC have been characterized as major determinants of the development of cognition in childhood (Bayliss et al., 2003) and old age (Park et al., 2002; Salthouse, 1994), and also of individual differences in intellectual abilities (Conway et al., 2003; Luck & Vogel, 2013; Vogel & Machizawa, 2004; Vogel et al., 2005). Understanding why WMC is finite is, therefore, an imperative step in understanding why cognitive abilities in humans are limited, why individuals have variability in these abilities, and how the abilities develop over the course of a lifetime (Oberauer et al., 2016). Discovering the neural mechanisms underlying such limitations is a central goal of cognitive neuroscience. Therefore, a robust and reliable behavior detection is essential for understanding the mechanisms behind WMC.

Encoding and retaining sensory stimulus sequences in WM is critical for adaptive behavior, especially for rodents (Fortin et al., 2002; Kesner et al., 2002). A fundamental challenge for the brain is to maintain as many items as possible in an active and recognizable state, while also preserving their temporal sequences (Buschman et al., 2011; Störmer et al., 2014). The WMC is limited, and a restricted amount of information or items can be saved at once. However, the procedures used to measure WM in rodents do not show limited memory capacity, but ceiling effect, which means high accuracy in mice or high percent correct in rats (April et al., 2013; Dudchenko et al., 2000; MacQueen et al., 2011). Thus, we developed a novel behavioral paradigm (Huang et al., 2020), the olfactory working memory capacity (OWMC) task, for detecting memory capacity in mice in a trial‐specific manner. We used the mice's sharpest sense, their sense of smell, to identify different odors. Then, we assessed the mice's ability to remember multiple odors based on nonmatch‐to‐sample rule. In this protocol, the sample odor for each trial is independent of the previous trial, and mice not only need to maintain more and more information from the list of odors, but also need to be flexible in using this information to respond appropriately. In the following, we will describe how to apply the paradigm to measure memory capacity.

## 2 MATERIALS AND METHODS

### 2.1 Mice

We used adult male heterozygous 5×FAD mice (overexpressing K670N/M671L + I716V + V717I mutations in human APP and M146L + L286V mutations in human PS1), which were purchased from Jackson Laboratory (Bar Harbor, ME, USA, strain no. 008730) (Jiang et al., 2020). And the wild‐type (WT) littermates were used as controls. The mice are raised in a temperature‐and humidity‐controlled environment (22 ± 2°C, 40−70%) with a 12‐h light/12‐h dark cycle. Food and water are freely available before the experiment. Age (3‐month) and body weight were kept matched among experimental control factors. During the training and testing period of the behavioral experiment, mice are fed 70–80% of their standard daily intake at the end of each day's experiment to maintain their body weight at around 80%. And the mice can drink freely. To effectively control the amount of food consumed by the mice each day, each mouse is kept in a separate home cage (32.5 cm long × 21 cm wide × 18 cm high). The use of rodents in experiments must comply with local and national regulations. All experimental animal procedures were approved by the Animal Care and Use Committee of the animal facility in Beijing.

### 2.2 Equipment

#### 2.2.1 A transparent Perspex training cage

**FIGURE 1:**
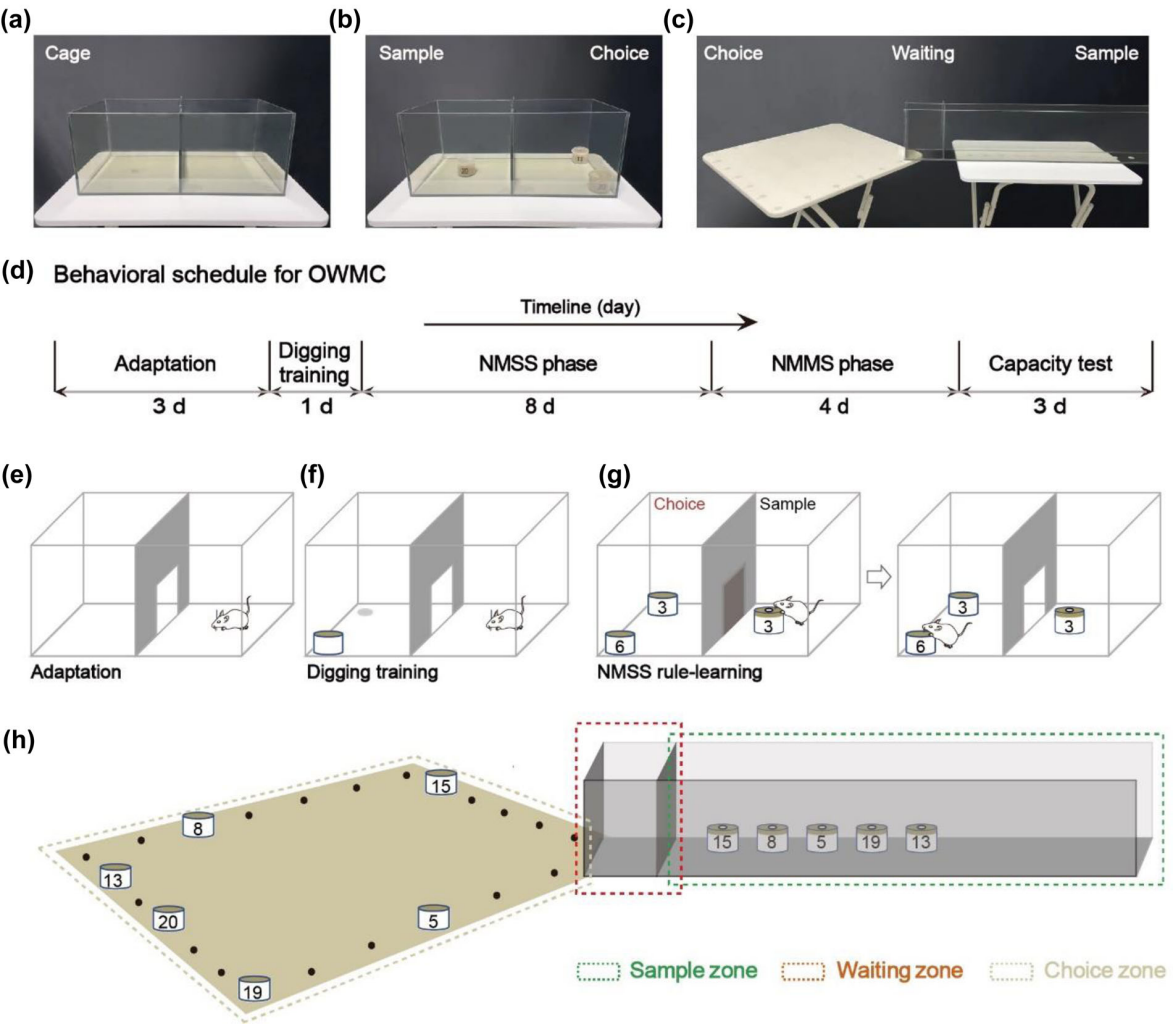
Training cage and platform. (a) The training cage for context adaptation, digging training, and NMSS rule‐learning. (b) Experimental design for the NMSS rule‐learning phase. (c) The spliced multipartition platform for NMMS rule‐learning and capacity testing. (d) Timeline of the OWMC task. (e–g) The context adaptation, digging training, and NMSS rule‐learning phase of the task. (h) The NMMS learning phase and capacity testing phase of the task

The transparent Perspex training cage is 46 cm long, 23 cm wide, and19 cm high. It consists of two chambers, separated by a manual side‐sliding door (20 cm long × 19 cm wide). The context adaptation, digging training, and nonmatching to a single sample odor (NMSS) rule‐learning take place in it (Figure 1a). The door is opened in context adaptation and digging training phases. In the NMSS rule‐learning phase, one chamber of the cage is designated as the sample chamber, and another is designated as the choice chamber (Figure 1b).

#### 2.2.2 A spliced multipartition platform

The NMMS rule‐learning and capacity testing take place on a spliced multipartition platform (Figure 1c). The platform consists of a square wooden table and a transparent Perspex passageway (connected to the table), divided by two manual side‐sliding doors into three zones, the sample zone, the waiting zone, and the choice zone (square wooden platform). The transparent Perspex passageway is 105 cm long, 10 cm wide, and 19 cm high. The passageway is divided into a sample zone (90 cm long) and a waiting zone (15 cm long), with a manual guillotine door (9.5 cm wide × 19 cm high) on either side of the waiting zone. The locations in the sample zone are numbered from 1 to 10 along with the chamber, and the locations are equally spaced. The square wooden platform is 61 cm long, 61 cm wide, and 71 cm high. Numbers 1–24 surround the platform's perimeter in sequence, with 1, 7, 13, and 19 at the four corners.

#### 2.2.3 Odor bowl

Each odor bowl contains a mixture of 7 g of sawdust, 1 g of cheese powder, and 0.5 g of the corresponding test odor (dill, cinnamon, chili, thyme, onion, rosemary, cumin, allspice, clove, almond, mint, matcha, basil, curry, ginger, caraway, coffee, celery, white pepper, and spinach) (Figure 1b). The bowls are 5.5 cm in diameter and 3.5 cm high, and each is marked with a number to identify the odor inside. A lid with a 1.5 cm diameter hole is placed over the sample odor bowl. Each bowl has a Velcro on the bottom and complementary Velcro dots are placed on the training cage, sample area, and platform to allow the bowls to be held in place.

### 2.3 Experimental design

This protocol for assessing OWMC consists of five phases (Figure 1d): context adaptation (3 days), digging training (1 day), NMSS rule‐learning (8‐12 days), NMMS rule‐learning (4‐8 days), and capacity testing (3‐5 days). The first phase is context adaptation, mice are handled to reduce stress and habituated to the training cage. In the digging training phase, mice are trained to dig up a grain of cheese in a bowl with unscented sawdust. Later, in the NMSS phase, mice are trained to dig for cheese pellets in the scented bowls with novel odor. Then, mice are trained to identify the novel odor from multiple scented bowls and to dig for the cheese pellet, which is the NMMS rule‐learning phase. Finally, capacity test, the mice will receive several sessions of WMC testing until they reach a stable level of performance. Below, we describe each phase in detail and list some critical steps to keep in mind in Table S1.

### 2.4 Context adaptation

Three days before the start of the experiment, the mice begin to be housed individually. And their body weight and food intake are recorded regularly every day, so as to calculate the standard daily food intake. For the first 3 days of the experiment, the mice are handled for 1–2 min to eliminate their fear of the operator and are placed in the training cage for 10 min of free movement to become fully familiar with the cage (Figure 1e).

### 2.5 Digging training

In this phase, mice are trained to dig up a grain of cheese (about 0.05 g) in a bowl with unscented sawdust for six trials, 1 day. Place an unscented sawdust bowl with a cheese pellet (in the center) at a corner of one chamber of the training cage, and then put the mouse from another chamber with its back onto the wall. During this stage, the center door is open, allowing the mice to explore freely and find the cheese pellets (Figure 1f). The cheese pellets are half‐buried in sawdust in the first three trials. And in the next three trials, they are buried under about 0.5 cm of sawdust in the same position.

### 2.6 NMSS rule‐learning

During the NMSS phase, the mice are trained to find cheese pellets in the scented bowls with novel odor by nonmatching principle. The sample odor bowl is placed in one chamber (the sample chamber) of the training cage, and a bowl with the matching (same) odor and another bowl with a nonmatching (novel) odor are placed in another chamber (the choice chamber) (Figure 1b). A grain of cheese is buried in the novel odor bowl. In each trial, mice are initially placed in the sample chamber, allowing for exploration and detection of the sample odor. Once the mouse sticks its nose into the hole of the lid, we assume that the mouse will investigate the odor. Then, the center door will be opened. Mouse is allowed to enter the choice chamber, investigating the two bowls (Figure 1g). The door is closed after the mouse enters the choice chamber. The mouse, if investigates the novel odor bowl and digs up the cheese pellet, after eating, will be put back in home cage to await another trial. If a mouse explores the same odor bowl and digs in, it is also briefly released back into its home cage and the positions of the two bowls are reassigned in a pseudo‐random manner. This trial is then repeated to ensure that the mice can only retrieve the pellets in novel odors and that incorrect responses are not rewarded. At the end of per trial, the training cage is scrubbed with disposable paper towels and 75% ethanol to reduce residual odors. The strict definition of “response” is that the mouse's forepaws or snout makes physical touch with the sawdust, coupled with the action of digging. During this phase, the inter‐trial interval (ITI) is 60 s. In this phase, the mice will receive 8−12 sessions, one session per day. Each session consists of 10 trials in which 20 different odors are randomly divided into 10 pairs of sample odors and novel odors so that the mice can be exposed to 20 odors every day. This stage consists of a minimum of eight sessions to ensure that the performance rate per mouse reaches at least 80% criteria. Then, the mice move on to the next stage (NMMS rule‐learning). If mice do not meet the standard after eight sessions, they will be trained for four more. At the end of this phase, the mouse that failed to reach the criteria of 80% in the 12th session will be excluded.

**FIGURE 2:**
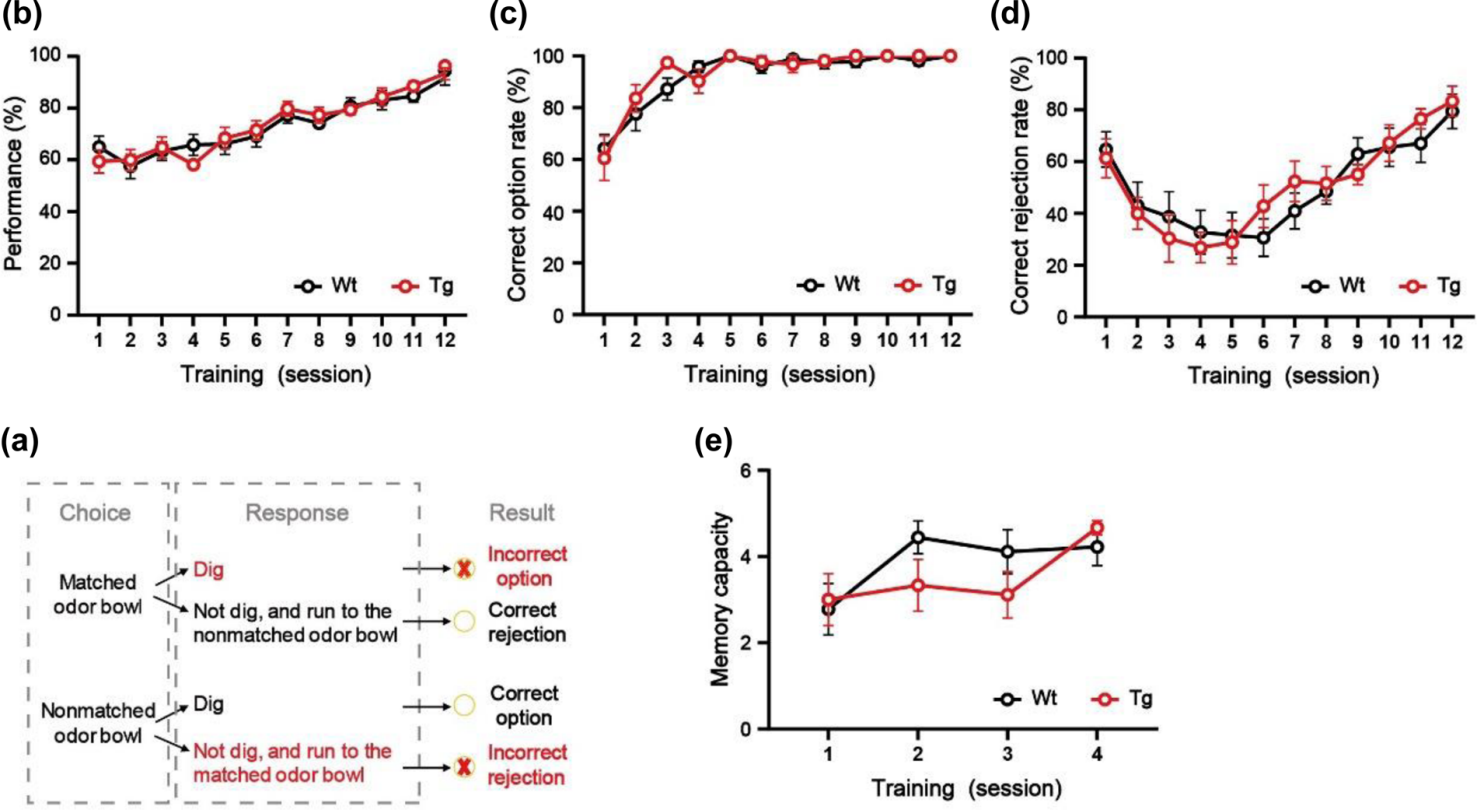
Mice learned the NMSS and NMMS rules well. (a) The different types of responses in the NMSS phase. (b–d) The performance correct rate, correct option rate, and correct rejection rate of mice during the NMSS‐rule leaning phase. (e) The memory capacity of mice during the NMMS‐rule leaning phase. *n* = 9 for all groups. Data are presented as the mean ± SEM.

Refer to the signal detection theory and previous studies, we introduced the parameters of “correct option,” “incorrect option,” “correct rejection,” “incorrect rejection,” and “omission” to analyze behaviors precisely during the training. The behavioral outcomes depend on the response to the first odor encountered. Each trial may produce four outcomes (Figure 2a): (1) when the mouse first encounters a nonmatching odor and digs up the cheese pellet, defined as correct option; (2) when the mouse first encounters a nonmatching odor, does not dig, turns to the matching odor, and has a digging action, defined as incorrect rejection; (3) if the mouse first encounters the matching odor and has the digging action, it is defined as incorrect option; and (4) if the mouse first encounters the matching odor but does not dig, it turns to the nonmatching odor and digs out the cheese pellet, that is correct rejection. If the mouse does not respond within the time limit (5 min), it will be defined as an omission. The definitions of performance correct rate, correct option rate, incorrect option rate, and correct rejection rate per session are shown in Table 1.

**TABLE 1:** Parameters for NMSS rule‐learning

**Performance correct rate = ** (No. of correct option trials + no. of correct rejection trials) / total number of trials %
**Correct option rate = ** No. of correct option trials / (no. of correct option trials + no. of incorrect rejection trials) %
**Incorrect option rate = ** No. of incorrect option trials / (no. of incorrect option trials + no. of correct rejection trials) %
**Correct rejection rate = ** No. of correct rejection trials / (no. of incorrect option trials + no. of correct rejection trials) %

### 2.7 NMMS rule‐learning

After reaching the 80% criteria during the NMSS rule‐learning phase, mice are introduced to the NMMS rule‐learning phase. Prior to the first trial, mice are allowed to familiarize the device's path twice, for approximately 2 min each time. In the first trial of per NMMS‐rule learning session (with nonmatching to one sample odor), mice are placed in the sample area to detect the sample stimulus, and then allowed to enter the waiting zone. After spending 5 s in the waiting zone, mice are allowed to pass through to select between a sample odor bowl and a novel odor bowl in the choice zone. A covered sample odor bowl is placed in the sample area, while a sample odor bowl and a novel odor bowl are placed in randomly specified locations in the choice area. Once the mouse starts digging in either bowl, the timer stops. If the mouse makes the right choice (digging into the nonmatching odor bowl), it will be given time to eat the reward food, then be returned to home cage until the next trial. When the mouse makes the wrong choice (digging into the matching odor bowl), this trial is immediately halted, and the mouse is placed back. The positions of bowls in the choice zone are randomly reassigned, and the trial is repeated until the mouse makes a correct response. During the second trial (with nonmatching to two sample odors), two additional novel sample odors are randomly selected from the odor stimulation pool and placed in the sample zone. The odor bowls for choice consist of two sample odor bowls and a novel odor bowl, placed in the choice area. When a mouse has sampled all odors, it can enter the waiting zone. Then, it enters the choice area to find the scent bowl where the food is hidden. This process is repeated additional three trials, adding one sample odor bowl for each trial. And in the fifth trial, there are five sample odor bowls (Figure 1h). The ITI is set for 3 min. This stage consists of 4−8 sessions, one session per day. At the beginning of per session, sample and choice odors for each of five trials are randomly chosen from 20 different odors, ensuring that per mouse is exposed to all 20 odors regularly over the phase of training. The NMMS training is repeated at least four sessions, making sure in two consecutive sessions each mouse completes nonmatching to two sample odors. If the mice fail, they will receive four more sessions. In additional training, the mice that still have not completed the trial of nonmatching to two sample odors for two sessions in a row scored 1 for their memory capacity.

### 2.8 Capacity testing

After the mice reliably complete the NMMS rule‐learning phase, they begin to undergo 3–5 sessions of WMC testing. This process is similar to the rule‐learning phase of the NMMS, where the mice have to remember an increasing number of sample odors. If two consecutive wrong choices are made, the experiment is terminated, and the mouse's capacity is scored as (*n* − 1). For example, if a mouse consistently responds incorrectly to a capacity level of 5 (five sample odors are presented in the trial), then the mouse's capacity score is 4. The correct and wrong responses are recorded, and the average error and correct rate are calculated to reflect the correct performance rate of the mice at each capacity level. The percentage of mice that could still successfully complete OWMC task at each level is calculated to compare the OWMC in different genotypes. And the chance level is the expected level of random selection, compared to the percent correct. When the number of sample odors (*n*) increases, the chance of correct selection (1/ (*n* + 1) %) decreases, and the chance level means the difficulty of the OWMC task. The parameters for the capacity test are shown in Table 2.

**TABLE 2:** Parameters for capacity testing

**Percent correct = ** No. of corrected trials at each capacity level / (no. of corrected trials + no. of all errors made by mice) %
**Percentage of mice that succeeded at each capacity level = ** No. of mice that succeeded in the trial at each capacity level / no. of mice in the test %
**Averaged errors = ** No. of all errors made by mice at each capacity level / no. of mice in the test

### 2.9 Statistical analysis

All data were presented as mean ± SEM, and statistical tests were performed using GraphPad Prism version 9.0 software. We used two‐tailed paired Student's *t* test to analyze the memory capacity of capacity test, and other data were analyzed by two‐way ANOVA. *p* < .05 was regarded as significant threshold in all experiments.

## 3 RESULTS

In the NMSS rule‐learning phase, to quantify the learning performance of the mice, we counted the performance correct rate, correct option rate, and correct rejection rate. As the number of training days increases, the learning performance of the mice will become better and better, showing the continuous improvement of three indicators. In our experiments, the mice were able to achieve a correct performance rate of 80% or more within 12 training sessions, and then all proceeded to the next rule‐learning phase. During the NMMS rule‐learning phase, the number of sample odors remembered by the mice gradually increased to about five after four training sessions. In the final test phase, the memory capacity of mice will tend to a stable fluctuating range after 3–5 consecutive tests. As the memory load increases, the average number of errors will increase, while the correct rate will keep decreasing. And, we also found that as the memory load and difficulty increased, the number of mice that succeeded in more difficult trials gradually decreased. It is appreciated that the novel paradigm measures the stability limit of OWMC in the mice, which is consistent with the results obtained in human WM tasks.

**FIGURE 3:**
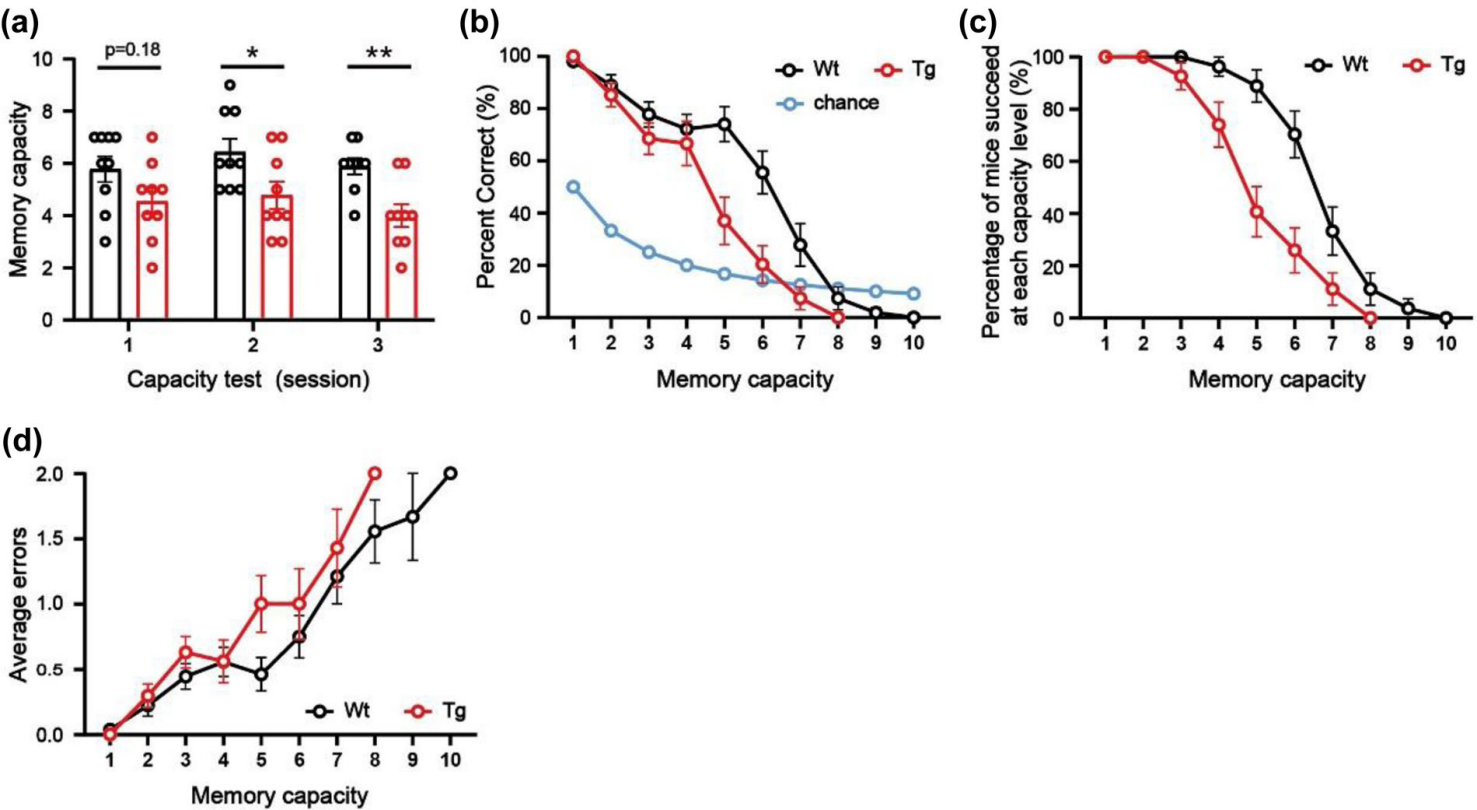
The WMC of 5 × FAD mice was significantly impaired. (a) The WMC of mice during the test phase (*t* test, *p < .05; **p < .01). (b) The percent correct at each capacity level (two‐way ANOVA, Tg vs. Wt: ****p < .0001). (c) The percentage of mice succeeded at each capacity level (two‐way ANOVA, Tg vs. Wt: ****p < .0001). (d) Average errors at each capacity level (two‐way ANOVA, Tg vs. Wt: *p < .05). *n* = 9 for all groups. Data are presented as the mean ± SEM.

For the application, we tested the OWMC of 3‐month‐old 5×FAD mice. In the NMSS rule‐learning phase, there was no difference between WT mice and 5×FAD mice in the performance correct rate, correct option rate, and correct rejection rate (all *p *> .05, Figure 2b‐d). After 4 days of NMMS rule‐learning, the WT mice and 5×FAD mice showed similar memory capacity (*p *> .05, Figure 2e). It is suggested that 3‐month‐old 5×FAD and WT mice can learn NMSS and NMMS rules well. After capacity test, we found that the WMC was significantly diminished in 5×FAD mice, compared to the littermate WT mice (day‐1: *t *= 1.47, *p *> .05; day‐2: *t *= 2.36, *p *< .05; day‐3: *t *= 4.15, *p *< .01, Figure 3a). As the memory load increased, 5×FAD mice made significantly more errors than WT mice (*F*(1, 326) = 5.41, *p *< .05, Figure 3d), had a significantly lower percent correct (*F*(1, 520) = 25.21, *p *< .0001, Figure 3b). And the mice of 5×FAD were able to complete the task at a significantly lower rate than WT mice (*F*(1,520) = 44.19, *p *< .0001, Figure 3c).

## 4 DISCUSSION

A plenty of procedures have been used to test WM in rodents, including the WM version of the Morris swimming task, the radial arm maze, and various delayed match‐ and nonmatch‐to‐sample tasks. Although memory capacity is a key component of WM, few studies have used WM tasks in rodents to explore the upper limit of memory capacity (Dudchenko et al., 2013). We derived the OWMC protocol from human span task and the rodent odor span task (OST) established by Dudchenko et al. (2000) and Young et al. (2007). In the version of the OST procedure, rodents are instructed to apply a rule (nonmatch‐to‐sample odor) to discern the novel odor among several odors, which has not been presented in the previous trial. During the session, the number of odor stimuli to remember increases, and the percent correct and span length (number of consecutive correct responses) are used to define the WMC (April et al., 2013; Kesner et al., 2002; Turchi & Sarter, 2000). But even with a high load, the performance of subjects has still shown a high accuracy and appears to remain well above chance level in OST (Dudchenko et al., 2000; MacQueen & Drobes, 2017; Young et al., 2009), while it has demonstrated a significant decline in human research (Nour et al., 2019; Proskovec et al., 2019). Considering that the classical WMC procedures for humans typically require continuous recall of the items, being remembered, they presented during the trial are only relevant to control behavior during a single trial (Pardo‐Vázquez & Fernández‐Rey, 2008; Schroeder et al., 2012). We developed the novel OWMC procedure in which we introduced a measure of WMC for trial‐specific information, and then we assessed the performance of mice in the paradigm from low to high load olfactory stimuli. Specially, in the previous OST procedure, the sample odors in per trial that subjects remembered comprised the types of odors, presented in previous trials, so rats or mice may remember certain types of odors repeatedly (April et al., 2013; Kesner et al., 2002). A major design of our protocol is the introduction of the NMMS phase, in which the sample odors in each trial are independent of those in previous trials. It requires the maintenance of information within a trial, in which subjects are not only required to maintain increasing amounts of odor information, but also required to use the information flexibly to respond appropriately.

The olfactory‐based WMC paradigm offers a series of general features that make it particularly suitable for studying the neurobiological basis of learning and memory processes. Over other methods of measuring WM, this paradigm has several advantages. First, the OWMC task, built upon a five‐stage protocol (context adaptation, digging training, NMSS, NMMS, and capacity testing), is designed to optimize performance across variable odors, including olfactory discrimination ability and WM. Second, the capacity test will be implemented in different zones, including encoding zone, waiting zone, and choice zone, allowing for reliable assessment of WMC, and underlying processes, including encoding, retention, and retrieval. Third, the OWMC task can also be designed to explore performance across variable retention delays, thus enabling the reliable assessment of memory decay and underlying processes of forgetting. Fourth, the paradigm can be easily implemented in a typical rodent facility by personnel with standard animal behavioral expertise.

The main disadvantage is that it requires longer training time due to the complex cognition process. Besides, one of the disadvantages is that the mice were deprived of food to maintain 70−80% of normal weight during training and testing for behavioral experiments. To minimize contributions from the confounding factors, mouse age, weight, and daily food intake should be matched through habituation and training procedures. Moreover, in mutant or manipulated mice, defects in the olfactory can also affect the OWMC test results. We assessed 3‐month‐old 5×FAD mice, which at a pretty young age, and mice could discriminate odor differences during the NMSS and NMMS phases. Consistently, previous studies indicated that 6‐month‐old 5×FAD mice have no olfactory deficits compared with their WT controls in olfactory detection (Roddick et al., 2016). In addition, 2‐ to 6‐month‐old 5×FAD mice showed no deficits on an olfactory maze task, indicating that the 5×FAD mice were able to detect the odors (Girard et al., 2014). So, we concluded that 3‐month‐old 5×FAD mice and WT mice do not differ in their ability to discriminate odors. Furthermore, the motivational state eager to acquire the rewards can alter the performance of the mice during the task. We found no relevant literature reporting that 3‐month‐old 5×FAD mice exhibit motivational deficits. And, in our experiments, we found that both WT mice and 5×FAD mice were full of craving for reward food. At the end of each day of the experiment, the food given to each mouse was quickly consumed. In addition, our experimental results also showed that there was no difference in NMSS and NMMS rule learning in 5×FAD mice compared to WT mice. Taken together, it shows that the 3‐month‐old 5×FAD mice did not show motivational impairment. Last, mice should be housed individually due to food deprivation protocols, which reduces social enrichment and may also have a slight effect on measures of cognition.

As a simple and easy‐to‐use method to analyze WMC, (1) the OWMC task can be used to investigate the neural mechanisms underlying WM. Impairment of WMC has been clinically identified in a variety of diseases, and the neurobiological mechanisms underlying the impairment of WM may differ across diseases. Using the OWMC paradigm, the cognitive impairment in the mice model of different diseases can be well detected and the neurobiological mechanisms behind them can be explored. (2) Clinically, early diagnosis and early treatment of multiple neuropsychiatric disorders are advocated. This paradigm can help identify early cognitive impairment in a variety of diseases (such as AD, schizophrenia, and autism), and can be used as a means to evaluate the effectiveness of pharmacological interventions or other intervention modalities. (3) The research design of the paradigm includes several aspects, such as information retrieval, information retention, and information extraction. We can use this paradigm to further explore the role of different processes in WM and neurobiological mechanisms that exist behind them. (4) Also, during the experimental design, we added a waiting zone, so that the performance under different retention delays can be artificially set to explore, and enabling the reliable assessment of memory decline and potential forgetting processes.

Overall, the OWMC task, based on a nonmatch‐to‐sample rule, is a sensitive and robust behavioral assay that we validated as a reliable method for measuring WMC. In addition, we list below some of the problems (Table 3) that may be encountered in the experiments, giving possible causes as well as solutions.

**TABLE 3:** Troubleshooting table

Phase	Problem	Possible reason	Solution
Digging training	The mice do not eat cheese.	Mice are not adapted to cheese as food.	Before the experiment began, the mice are allowed to sample a small piece of cheese, then start the formal experiment.
	Instead of looking for the reward food in the sawdust bowl, the mice wander aimlessly around the training cage.	Mice that are restricted from eating may not be as hungry.	Appropriately prolong the time mice are restricted from eating to enhance their desire for food.
NMSS rule‐learning	On the first day of this phase, when mice fail several times consecutively on a trial, they may become less motivated to search for reward food or even stop looking.	Mice that have not yet learned the rules may cling to a scented bowl if it is a bowl with the same odor, and they may assume that none of the scented bowls in the choice chamber have reward food.	It is recommended to pause this trial and try to start the next trial. Through positive feedback, the mice are made to understand that there is reward food in the bowl with novel odor in the choice chamber.
	Upon entering the choice chamber, the mice first explore the scent bowl with a possible location preference.	A search strategy developed by the mice themselves.	The bowls with the novel odors in the choice chamber, 10 trials per day, are equally distributed in two locations, that is, the novel odor bowls will appear five times in each location.
	For the 10 trials per day, when the mice reach the later trials, they stop looking for food, or their desire for food is significantly reduced.	The mice's hunger is diminished by feeding on the preceding reward food.	The reward food should not be too large.
NMMS rule‐learning	On the first day of this phase, mice become more nervous and spend more time on exploring the spliced multipartition platform.	At this stage, the behavioral apparatus is changed and the mice have not yet adapted to the new apparatus.	Before the experiment began, the mice are allowed to explore the apparatus twice for approximately 2 min each time.
Capacity testing	The WMC of mice is unstable.	The condition of the mice may vary from day to day, and the test situation may be slightly different, such as the position of the scent bowls on the square wooden platform.	It is recommended to increase the number of days of testing.

## COMPETING INTERESTS

The authors declare no competing interests.

## AUTHOR CONTRIBUTIONS

G.D.H., H.L.W., and X.Y. designed the experiments. L.X.J. optimized experimental procedures. L.X.J. performed the experiments. H.L.W., C.Z., and X.Y. commented on the manuscript. L.X.J., G.D.H., and X.Y. wrote the manuscript.

### PEER REVIEW

The peer review history for this article is available at https://publons.com/publon/10.1002/brb3.2703

## Supporting information

Table S1. Critical steps in different phase

## Data Availability

Data supporting the results of this study can be obtained from the corresponding authors upon reasonable request.
