# Supplementary material for: The protocol for assessing olfactory working memory capacity in mice
Source: Brain Behav. 2022 Jul 18;12(8):e2703. doi: 10.1002/brb3.2703 (PMC9392537; doi:10.1002/brb3.2703)
Supplement: Supplementary file 1 — Table S1. Critical steps in different phase [file BRB3-12-e2703-s001.docx]

**Table S1.** Critical steps in different phase

| **Context adaptation**   - In order for mice to have sufficient cravings for the reward food in subsequent experiments, they need to be food restricted. Therefore, the standard daily food intake of mice needs to be specified before the start of the experiment.   **Digging training**   - For 6 trials, bowls are randomly placed 3 times in each of two corners to avoid the mice associating food reward with location. In the first 3 trials, the food needs to be half-buried in sawdust, not all, or the mice may not know there is food in the bowl. And keep the food in the same position, with the center of the bowl about 0.5 cm deep.   **NMSS rule-learning**   - A perforated lid needs to be added to the sample scent bowl so that the mouse can smell the scent inside, but cannot dig. - When the mouse digs up the reward food, wait for it to finish eating and then put it back in the home cage. In this way, the mice are allowed to establish rewarding feedback that keeps them sufficiently eager. - When the mouse responds incorrectly, it should be taken out of the training cage immediately. The early stage of training, a critical period for behavior shaping and rule learning, requires maintaining sufficiently strict operating standards. - For 10 trials per day, the novel scent bowls are randomly placed in each of the two corners 5 times. 20 different odors are randomly assigned to 10 pairs of sample odors and novel odors, ensuring that the mice are exposed to each scent on a daily basis.   **NMMS rule-learning**   - The mice need to have sniffed all sample odors before they can enter the waiting zone.   **Capacity testing**   - When the mice make two consecutive wrong choices, the experiment ends. |
| --- |
